# The antimicrobial activity of zinc against group B *Streptococcus* is strain-dependent across diverse sequence types, capsular serotypes, and invasive versus colonizing isolates

**DOI:** 10.1186/s12866-021-02428-3

**Published:** 2022-01-13

**Authors:** Jamisha D. Francis, Miriam A. Guevara, Jacky Lu, Shabir A. Madhi, Gaurav Kwatra, David M. Aronoff, Shannon D. Manning, Jennifer A. Gaddy

**Affiliations:** 1 Department of Pathology, Microbiology and Immunology, Vanderbilt University Medical Center, Nashville, TN 37212 USA; 2 South African Medical Research Council Vaccines and Infectious Diseases Analytics Research Unit, Faculty of Health Sciences, University of the Witwatersrand, Johannesburg, South Africa; 3 Department of Clinical Microbiology, Christian Medical College, Vellore, India; 4 Department of Medicine, Division of Infectious Diseases, Vanderbilt University Medical Center, A2200 Medical Center North, 1161 21st Avenue South, Nashville, TN 37232 U.S.A.; 5 Department of Obstetrics and Gynecology, Vanderbilt University Medical Center Nashville, Nashville, TN 37232 USA; 6 Department of Microbiology and Molecular Genetics, Michigan State University, East Lansing, MI 48824 USA; 7 Department of Veterans Affairs, Tennessee Valley Healthcare Systems, Nashville, TN 37212 USA

**Keywords:** Antimicrobial, Metal, Zinc, *Streptococcus agalactiae*, Group B *Streptococcus*

## Abstract

**Background:**

*Streptococcus agalactiae* or Group B *Streptococcus* (GBS) is an encapsulated gram-positive bacterial pathobiont that commonly colonizes the lower gastrointestinal tract and reproductive tract of human hosts. This bacterium can infect the gravid reproductive tract and cause invasive infections of pregnant patients and neonates. Upon colonizing the reproductive tract, the bacterial cell is presented with numerous nutritional challenges imposed by the host. One strategy employed by the host innate immune system is intoxication of bacterial invaders with certain transition metals such as zinc.

**Methodology:**

Previous work has demonstrated that GBS must employ elegant strategies to circumnavigate zinc stress in order to survive in the vertebrate host. We assessed 30 strains of GBS from diverse isolation sources, capsular serotypes, and sequence types for susceptibility or resistance to zinc intoxication.

**Results:**

Invasive strains, such as those isolated from early onset disease manifestations of GBS infection were significantly less susceptible to zinc toxicity than colonizing strains isolated from rectovaginal swabs of pregnant patients. Additionally, capsular type III (cpsIII) strains and the ST-17 and ST-19 strains exhibited the greatest resilience to zinc stress, whereas ST-1 and ST-12 strains as well as those possessing capsular type Ib (cpsIb) were more sensitive to zinc intoxication. Thus, this study demonstrates that the transition metal zinc possesses antimicrobial properties against a wide range of GBS strains, with isolation source, capsular serotype, and sequence type contributing to susceptibility or resistance to zinc stress.

## Introduction

Group B *Streptococcus* (GBS), or *Streptococcus agalactiae*, infections are one of the top five leading causes of neonatal mortality. GBS infection induces chorioamnionitis, preterm prelabor rupture of the gestational membranes (PPROM), preterm birth, and both maternal and neonatal sepsis [1]. GBS disease in neonates often manifests as early-onset or late-onset sepsis, pneumonia, or meningitis and can lead to death [2].

GBS is an encapsulated gram-positive bacterium that colonize the urogenital and/or lower gastrointestinal tract of healthy women and colonization rates vary between 15 and 40% depending on geographical region [3–5]. During infection of the upper female reproductive tract, GBS ascends the vagina to the cervix and then to the uterus where the bacteria can cross the gestational membrane barrier. GBS infection of these extraplacental, gestational membranes (i.e., chorioamnionitis) can provoke inflammation that triggers labor and/or causes PPROM [1]. Infection of the fetus by GBS can also lead to stillbirth or neonatal sepsis. GBS can cause early-onset neonatal disease (EOD) (within the first week of life), of which 90% occurs 0-3 days after birth, or late-onset disease (LOD), which occurs one week to three months after birth [2].

Current recommendations for prevention of EOD focus on maternal GBS screening at 35-37 week’s gestation and the use of intrapartum antibiotic prophylaxis (IAP). Beta-lactam antibiotics such as penicillin and ampicillin, are used in GBS positive expecting mothers [6]. Currently, IAP has not been as effective for the treatment and prevention of LOD, nor has it reduced the incidence of PPROM, preterm birth, or stillbirth [6, 7]. In addition, the rise in antibiotic resistant GBS strains is also of grave concern [8]. Thus, developing new interventions to control antibacterial resistance is of great importance. Subsequently, these innovations will decrease the incidence of maternal-fetal GBS infections and complications.

Zinc, copper, and iron are transition metals that are essential micronutrients for all cells. In both eukaryotes and prokaryotes, transition metals serve as co-factors for enzymes that perform critical cellular processes [9]. These metal ions, though required, can become toxic to bacteria at high concentrations [10]. The host exploits this specifically within innate immune cells, such as macrophages and neutrophils, which load the phagosome with divalent zinc cations (Zn^2+^) to intoxicate phagocytosed bacteria [11, 12]. To circumnavigate this, bacteria must regulate metal import and export machinery to maintain normal zinc levels for growth and survival [13].

Recent work demonstrates that GBS employs a repertoire of factors to facilitate metal homeostasis and promote bacterial survival in the vertebrate host. Specifically, GBS require efflux determinants to overcome metal stress and promote survival. Sullivan and colleagues reported that GBS elaborates the CzcD efflux system, activated by the SczA response regulator to manage intracellular zinc levels [14]. Furthermore, they demonstrated that the CzcD and SczA systems are critical for zinc resistance, survival within macrophages, and dissemination in a mouse model of invasive disease in the blood, heart, liver, and bladder. Together, these results indicate that zinc is an important innate immune antimicrobial strategy employed against GBS, and that GBS resistance to zinc toxicity is critical for full virulence.

While zinc has been implicated in modulating the immune response to infection [15], the role zinc plays in the context of perinatal-related GBS infections has not yet been elucidated. In this study, we advanced previous findings by analyzing the antimicrobial effects of zinc against a panel of clinical GBS strains that vary by capsular serotype, sequence type (ST), isolation source, and clinical presentation. We observed strain-specific variation in susceptibility to zinc intoxication with specific differences between invasive and colonizing strains and across different capsular serotypes and STs.

## Methods

### Bacterial strains and culture conditions

A bank of 27 GBS clinical strains were provided by Dr. H. Dele Davies following recovery from neonates with invasive disease [16] and colonized mothers sampled before and after childbirth [17] for use in this study (Table 1). These strains were all isolated from separate patients and were previously characterized using multilocus sequence typing and cps typing [18, 19]. Three common laboratory reference strains (A909, NEM316, and COH1; American Type Culture Collection) were also evaluated. Bacterial strains were cultured on tryptic soy agar (TSA) plates supplemented with 5% sheep blood at 37 °C overnight followed by inoculation into brain-heart infusion broth (BHI) and incubation in aerobic conditions (ambient air, shaking at 200 rpm) at 37 °C. After 24 h, bacterial density was measured spectrophotometrically to determine the optical density at 600 nm (OD_600_). These bacterial cultures were used for growth and viability assays.

**Table 1:** Strain identifier, strain type, Isolation source, capsular type, and sequence type (ST) of clinical strains of *Streptococcus agalactiae* used in this study and the minimum inhibitory concentration (MIC) of zinc chloride required to suppress growth (as determined by OD_600_) by comparison with growth in medium alone lacking zinc supplementation (*P* < 0.05, Student’s *t* test)

Strain Identifier	Strain Type	Sequence Type	Capsular Serotype	Isolation Source	Growth MIC
**GB0002**	Colonizing	ST-23	cpsIa	Vaginal/rectal colonization	2500 μM
**GB0012**	Colonizing	ST-1	cpsV	Vaginal/rectal colonization	500 μM
**GB0037**	Invasive	ST-1	cpsV	EOD/sepsis	1000 μM
**GB0064**	Invasive	ST-17	cpsIII	EOD/sepsis	2500 μM
**GB0066**	Invasive	ST-19	cpsIII	EOD/sepsis	> 5000 μM
**GB0069**	Invasive	ST-17	cpsIII	EOD/sepsis	2500 μM
**GB0079**	Invasive	ST-19	cpsIII	EOD/sepsis	> 5000 μM
**GB0083**	Colonizing	ST-1	cpsVI	Vaginal/rectal colonization	125 μM
**GB0112**	Colonizing	ST-12	cpsIII	Vaginal/rectal colonization	500 μM
**GB0115**	Colonizing	ST-17	cpsIII	Vaginal/rectal colonization	2500 μM
**GB0241**	Colonizing	ST-23	cpsV	Vaginal/rectal colonization	1000 μM
**GB0285**	Colonizing	ST-12	cpsII	Vaginal/rectal colonization	2500 μM
**GB0291**	Colonizing	ST-12	cpsII	Vaginal/rectal colonization	2500 μM
**GB0374**	Invasive	ST-12	cpsIb	EOD/sepsis	2500 μM
**GB0377**	Invasive	ST-19	cpsIII	EOD/sepsis	> 5000 μM
**GB0390**	Invasive	ST-23	cpsIa	EOD/sepsis	> 5000 μM
**GB0397**	Invasive	ST-23	cpsIII	EOD/sepsis	1000 μM
**GB0411**	Invasive	ST-17	cpsIII	EOD/sepsis	750 μM
**GB0418**	Invasive	ST-17	cpsIII	EOD/sepsis	2500 μM
**GB0438**	Invasive	ST-12	cpsIb	LOD/sepsis	1000 μM
**GB0561**	Colonizing	ST-19	cpsV	Vaginal/rectal colonization	125 μM
**GB0571**	Colonizing	ST-19	cpsIII	Vaginal/rectal colonization	250 μM
**GB0590**	Colonizing	ST-19	cpsIII	Vaginal/rectal colonization	> 5000 μM
**GB0651**	Colonizing	ST-19	cpsIb	Vaginal/rectal colonization	125 μM
**GB0653**	Colonizing	ST-12	cpsII	Vaginal/rectal colonization	1000 μM
**GB0654**	Colonizing	ST-17	cpsIII	Vaginal/rectal colonization	2500 μM
**GB0663**	Colonizing	ST-19	cpsIII	Vaginal/rectal colonization	> 5000 μM
**NEM316**	Invasive	ST-23	cpsIII	EOD/sepsis	500 μM
**COH1**	Invasive	ST-17	cpsIII	Blood	750 μM
**A909**	Invasive	ST-7	cpsIa	Blood/sepsis	125 μM

### Evaluation of bacterial growth

GBS growth was determined by a spectrophotometric reading of optical density (OD) at OD_600_ as previously described [20]. Briefly, GBS cultures were grown overnight and diluted at 1:10 in fresh BHI medium; 100 μL of 1:10 diluted cultures were added to each well in a 96-well plate. Increasing concentrations of divalent zinc ions (Zn^2+^) in the form of zinc chloride (ZnCl_2_) (0, 125, 250, 500, 750, 1000, 2500, 5000 μM) were added to the culture media. These concentrations were chosen because they represent a range of physiologically relevant concentrations often encountered in the host-pathogen environment in vivo [11]. The plates were incubated statically in 5% CO_2_ at 37 °C overnight. The following day, bacterial density was estimated via OD_600_. Three fresh biological replicates were assessed with 1-3 technical replicates within each biological replicate, and the OD_600_ values were normalized to a blank control of sterile, uninoculated bacteriological medium (BHI).

### Statistical analyses

Statistical analyses were performed using Mann-Whitney U for MIC studies, and either Student’s *t*-test or one-way ANOVA with either Tukey’s or Dunnett’s post hoc correction for multiple comparisons for bacterial growth assays. All reported *P* values were adjusted to account for multiple comparisons. *P* values of ≤0.05 were considered significant. All data analyzed in this work were derived from at least three biological replicates. Statistical analyses were performed using GraphPad Prism 9 software (GraphPad Prism Software Inc., La Jolla, California).

## Results

### High concentrations of zinc suppress bacterial growth in many clinical GBS isolates

Previous reports indicate that zinc has antimicrobial activities against GBS [14]. To enhance the generalizability of these findings, we sought to test a larger number of GBS strains, thereby capturing more isolates (both colonizing and invasive) across diverse capsular serotypes and genetic STs. We also investigated the effects of increasing zinc concentration exposure. Out of the 30 GBS strains screened, 4 strains (GB0083, GB0561, GB0651, and A909) exhibited significant inhibition of bacterial growth when treated with 125 μM zinc (Table 1; *P* < 0.05, Student’s *t* test, compared to medium alone control cultures). All of these strains were classified as colonizing strains except the single laboratory strain (A909). At 250 μM zinc, an additional colonizing strain (GB0571) exhibited significant inhibition of bacterial growth compared to cultures grown in medium alone (Table 1; *P* < 0.05, Student’s *t* test). Growth of three additional strains, two colonizing strains and one laboratory strain (GB0012, GB0112, and NEM316, respectively), was inhibited when treated with a concentration of 500 μM zinc (Table 1; *P* < 0.05, Student’s *t* test, compared to medium alone control cultures). At 750 μM zinc, strains GB0411 (invasive) and COH1 (laboratory) exhibited significant inhibition of bacterial growth compared to the medium only control (Table 1; *P* < 0.05, Student’s *t* test). The growth of 5 additional strains (GB0037, GB0397, GB0438, GB0241, GB0653), including three invasive and two colonizing isolates, respectively, was inhibited when treated with 1000 μM zinc (Table 1; *P* < 0.05, Student’s *t* test, compared to medium alone control cultures). The growth of 9 additional strains (GB002, GB0115, GB0285, GB0291, GB0654, GB0064, GB0069, GB0374, GB0418), including 5 colonizing and 4 invasive strains, respectively, was significantly inhibited when treated with 2500 μM zinc (Table 1; *P* < 0.05, Student’s *t* test, compared to medium alone control cultures). Finally, the growth of six strains (GB0066, GB0079, GB0377, GB0390, GB0590, and GB0663), including four invasive strains and two colonizing strains, respectively, was unaffected when treated with concentrations of zinc up to 5000 μM (Table 1; *P* > 0.05, Student’s *t* test, compared to medium alone control cultures).

### GBS colonizing and invasive strain types differ in susceptibility to zinc intoxication

**Fig. 1:**
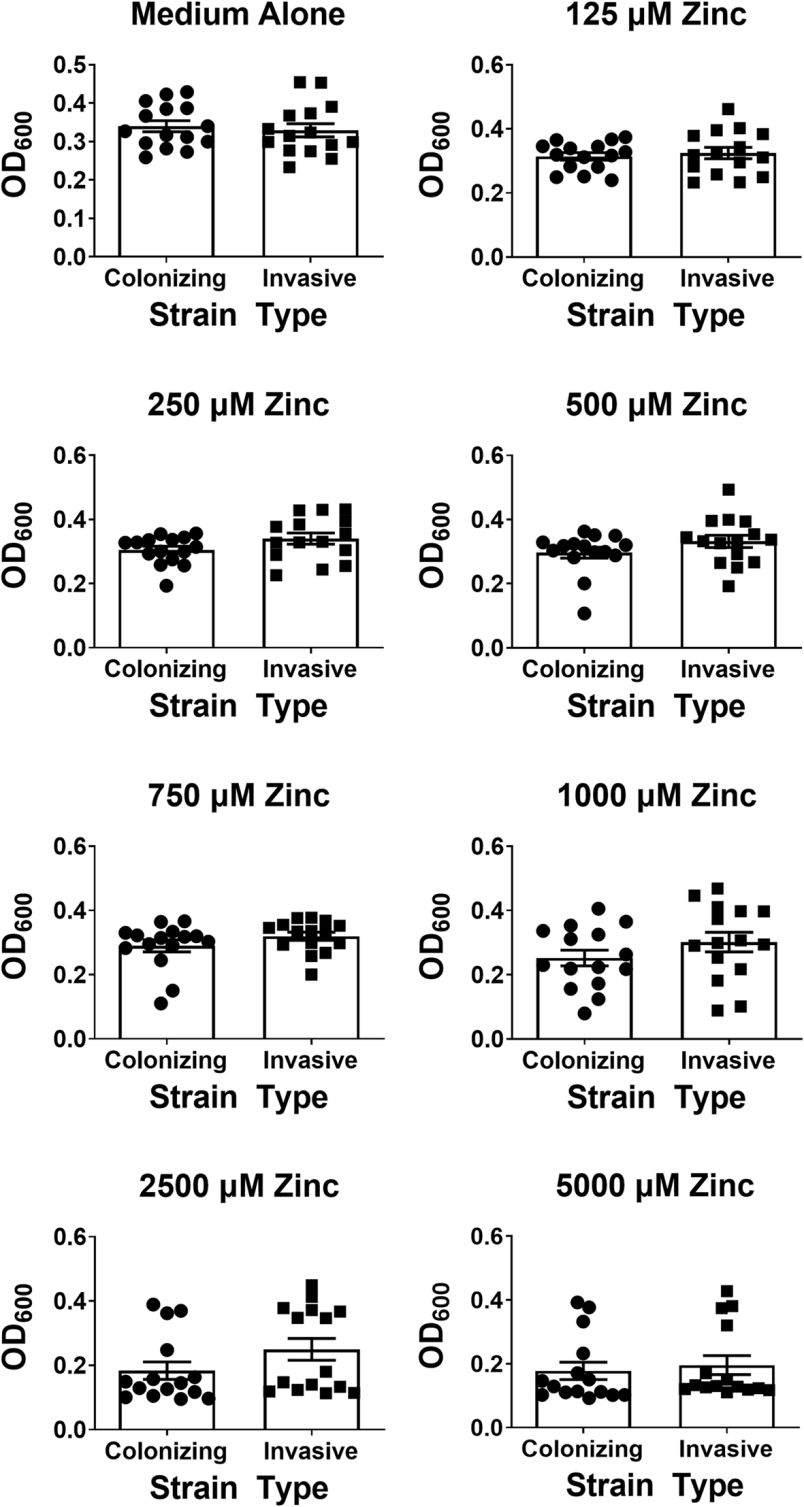
Analysis of susceptibility to zinc-associated growth inhibition in invasive vs. clinical isolates of Group B *Streptococcus* (GBS). GBS strains isolated from colonized patients, or patients experiencing invasive disease were grown in medium alone (Medium Alone) or increasing concentrations of zinc chloride (125 μM, 250 μM, 500 μM, 750 μM, 1000 μM, 2500 μM, 5000 μM). Bacterial growth was measured at 24 h post-inoculation as an optical density at 600 nm absorbance (OD_600_). At 0, 250, 500, 750, 1000, 2500, and 5000 μM concentrations of zinc, colonizing strains of GBS (circles) showed no significant differences in OD_600_ values across strain type

**Fig. 2:**
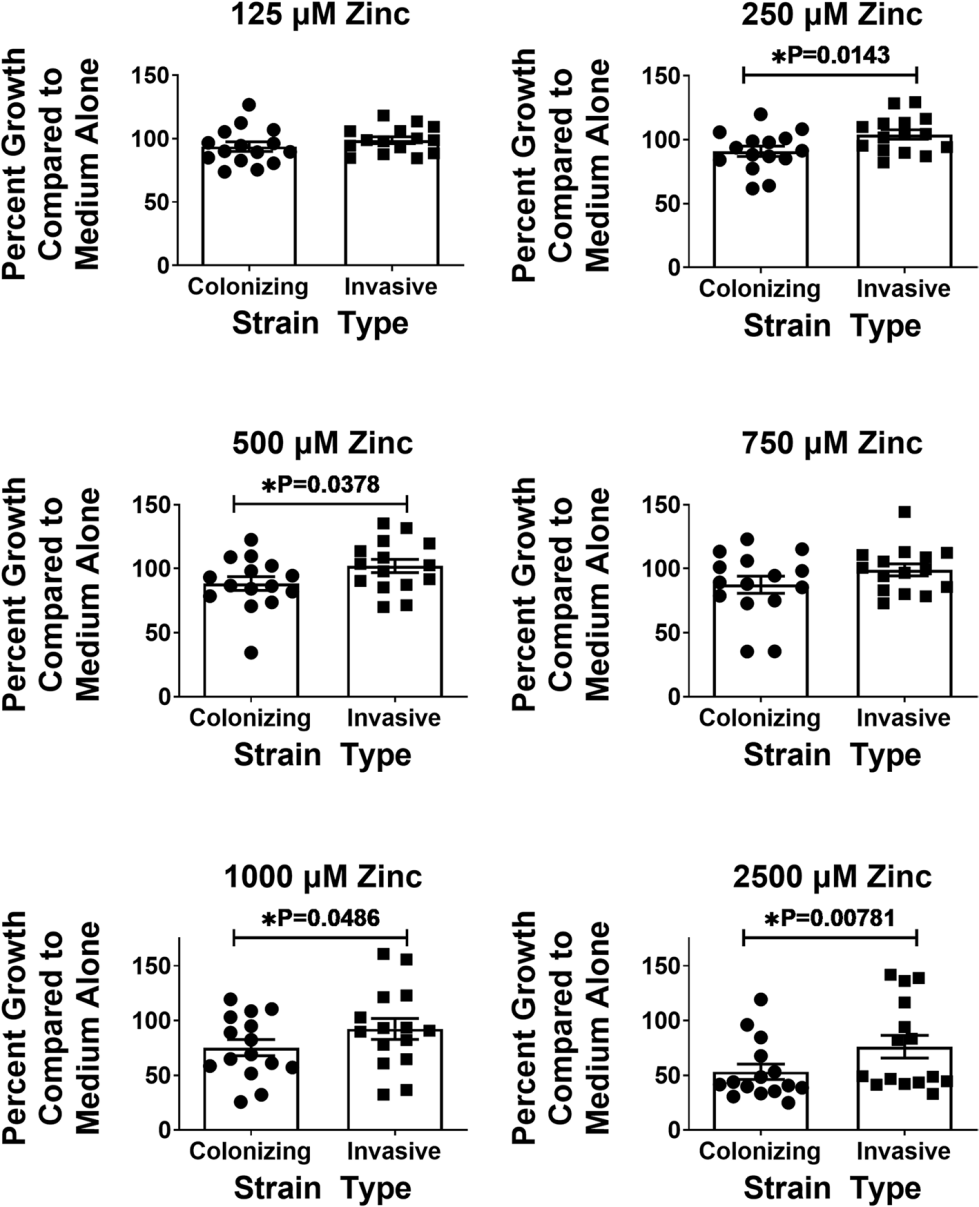
Analysis of percent growth of invasive vs. clinical isolates of Group B *Streptococcus* (GBS) when cultured under increasing concentrations of zinc. GBS strains isolated from colonized patients, or patients experiencing invasive disease were grown in medium alone (Medium Alone) or increasing concentrations of zinc chloride (125 μM, 250 μM, 500 μM, 750 μM, 1000 μM, 2500 μM). Bacterial growth was measured at 24 h post-inoculation as an optical density at 600 nm absorbance (OD_600_). At 250, 500, 1000, and 2500 μM concentrations of zinc, invasive strains of GBS (circles) showed significantly higher percent growth compared to medium alone (as calculated by mean percent growth of three biological replicates for each strain, comparing OD_600_ values in each zinc concentration compared to OD_600_ values for each strain in medium alone). Statistical significance was determined by paired Student’s *t* test (*n* = 3 biological replicates)

**Fig. 3:**
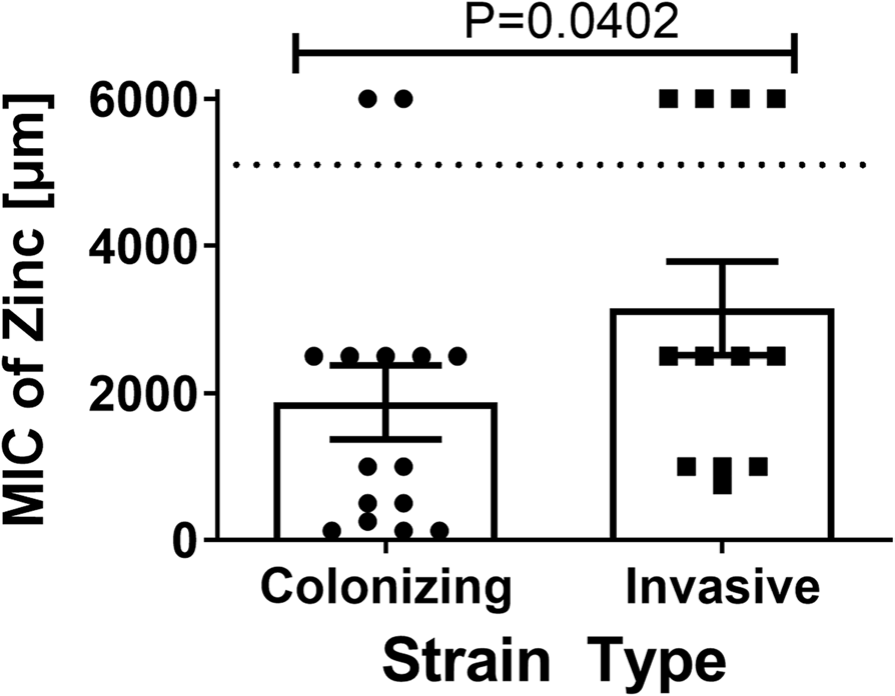
Minimum Inhibitory Concentration (MIC) of zinc chloride required to inhibit GBS growth across colonizing or invasive strains. Analysis of susceptibility to zinc-associated growth inhibition in invasive vs. clinical isolates of Group B *Streptococcus* (GBS). GBS strains isolated from colonized patients, or patients experiencing invasive disease were grown in increasing concentrations of zinc chloride and mean MIC was calculated. Significant differences were determined by Mann-Whitney U test (*P* < 0.05). Dotted line indicates upper limit of detection

Because zinc has been shown to be a crucial antimicrobial strategy deployed by the innate immune system [10–12], we hypothesized that there could be differences in susceptibility to zinc intoxication between colonizing and invasive GBS strains. To test this, we stratified the GBS strains by clinical phenotype. Strains were classified as “colonizing” if they were recovered from asymptomatic women sampled before or after childbirth, whereas “invasive” strains were isolated from babies with GBS disease. Strains were exposed to increasing concentrations of zinc chloride and growth was measured after 24 h of static incubation in 5% CO_2_ at 37 °C (Fig. 1). In medium alone, the mean OD_600_ measurement was calculated as 0.34 for colonizing strains and 0.33 for invasive strains, results that were statistically indistinguishable (*P* = 0.1627, Mann-Whitney U test). Similarly, no significant difference was noted in raw OD_600_ values between colonizing and invasive strains at 125, 250, 500, 750, 1000, 2500, or 5000 μM zinc exposure. However, calculation of percent growth of each strain (comparing growth of a specific strain at each zinc concentration compared to growth of that strain in medium alone) revealed that invasive strains exhibited a significantly enhanced mean growth when treated with 250, 500, 1000, and 2500 μM zinc (12, 12, 14, 19, and 36%, respectively) compared to colonizing strains (*P* < 0.05, Mann-Whitney U test, Fig. 2). Exposure to 250 μM zinc resulted in invasive strains having 13% higher percent growth compared to colonizing strains, a result that was statistically significant (*P* = 0.0143, paired Student’s *t* test; *P* = 0.0329, Mann-Whitney U test). Exposure to 500 μM zinc resulted in a 14% higher percent growth compared to colonizing strains (*P* = 0.0378, paired Student’s *t* test; *P* = 0.1160, Mann-Whitney U test). Exposure to 1000 μM zinc resulted in a 25% mean decrease in colonizing strain growth, compared to only a 9% in invasive strains (*P* = 0.0486, paired Student’s *t* test; *P* = 0.2328, Mann-Whitney U test). Exposure to 2500 μM zinc resulted in a 47% mean decrease in colonizing strain growth, compared to a 24% percent decrease in invasive strains (*P* = 0.00781, paired Student’s *t* test; *P* = 0.0555, Mann-Whitney U test). At a concentration of 5000 μM zinc, no significant difference in growth was observed between colonizing and invasive strains, largely because the growth of most strains was significantly inhibited at this concentration. Comparison of minimal inhibitory concentrations (MIC) of zinc to repress growth for colonizing versus invasive strains, revealed colonizing strains have a mean MIC of 1875 μM zinc, whereas invasive strains have a mean MIC of 3145 μM zinc (Fig. 3), a 68% increase which was statistically significant (*P* = 0.0402, Mann-Whitney U test).

### Susceptibility to zinc toxicity differs across GBS strains from varying isolation sources

**Fig. 4:**
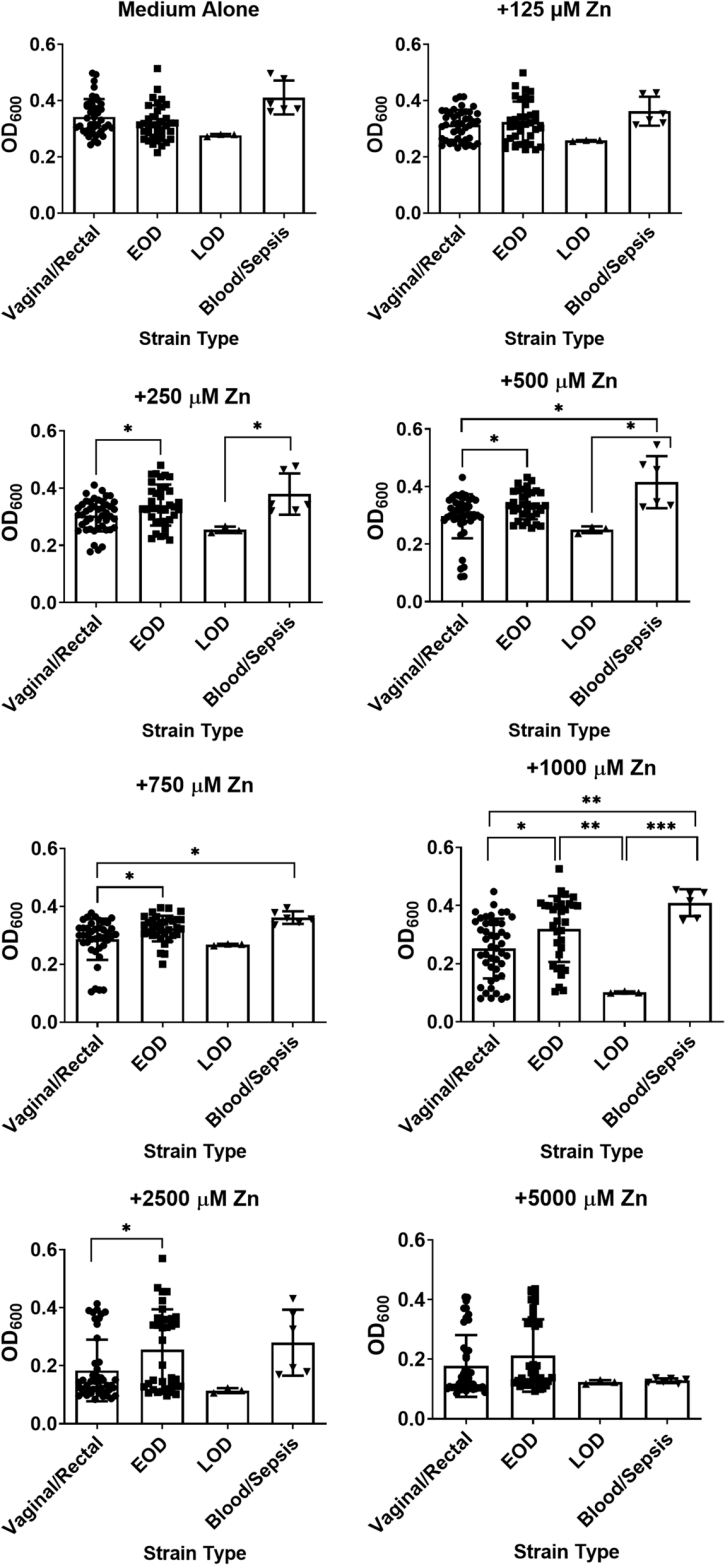
Analysis of susceptibility to growth inhibition by zinc intoxication based on isolation source. Group B *Streptococcus* (GBS) strains isolated from recto-vaginal swabs (Vaginal/Rectal, circles), early onset disease in neonates (EOD, squares), late onset disease in neonates (LOD, triangles), or blood/sepsis in adults (Blood/Sepsis, inverted triangles) were grown in medium alone (Medium Alone) or increasing concentrations of zinc chloride (125 μM, 250 μM, 500 μM, 750 μM, 1000 μM, 2500 μM, 5000 μM). Bacterial growth was measured at 24 h post-inoculation as an optical density at 600 nm absorbance (OD_600_). At 250, 500, 750, 1000 and 2500 μM concentrations of zinc, rectovaginal strains of GBS (circles) exhibited greater growth inhibition than strains isolated from early onset disease in neonates (squares), as determined by one-way ANOVA with Tukey’s post hoc multiple correction (*P < 0.05, and **P < 0.01, *n* = 3 biological replicates)

Because differences were observed between invasive and colonizing strains, we hypothesized that the source of bacterial strain isolation could contribute to zinc intoxication susceptibility due to GBS adaptation to the ecology of the specific host niche. To test this, we stratified strains into categories based on source of strain isolation (Fig. 4) such as blood from neonatal early onset disease (EOD), late onset disease (LOD), rectal or vaginal swabs (Vaginal/Rectal), or adult blood from septic patients (Blood/Sepsis). Results indicate that at concentrations of 250, 500, 750, 1000, and 2500 μM zinc, isolates from EOD have significantly enhanced growth (12, 13, 13, 27, and 39%, respectively) compared to vaginal/rectal isolates (*P* < 0.05, one-way ANOVA). At concentration of 250 μM zinc, LOD isolates were significantly more susceptible to zinc toxicity than those isolated from blood/sepsis (*P* < 0.05, one-way ANOVA). At 500 μM zinc, blood/sepsis isolates were significantly more tolerant of zinc stress than their vaginal/rectal or LOD isolate counterparts (*P* < 0.05, one-way ANOVA). At 750 μM zinc, blood/sepsis and EOD isolates were significantly less susceptible to zinc stress than rectovaginal isolates. At 1000 μM zinc, EOD and blood/sepsis isolates were significantly less susceptible than rectovaginal isolates. However, GBS isolated from LOD were significantly more susceptible than strains from the other three isolation sites.

### GBS capsular serotype confers varying susceptibility to zinc intoxication

**Fig. 5:**
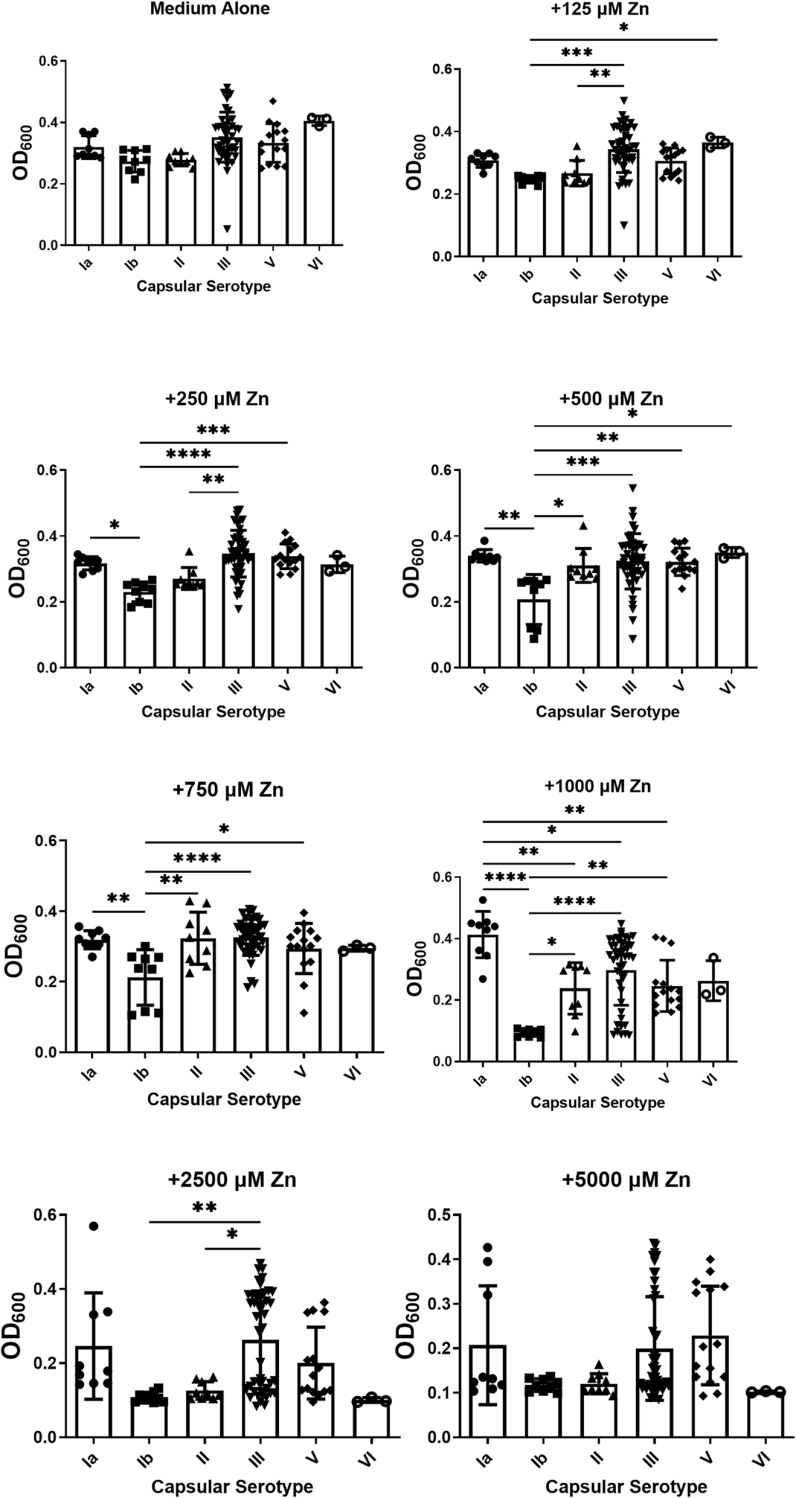
Analysis of susceptibility to growth inhibition by zinc intoxication in diverse capsular serotypes of Group B *Streptococcus* (GBS). GBS strains isolated with a span of capsular serotypes (cpsIa, black circles; cpsIb, squares; cpsII, triangles; cpsIII, inverted triangles; cpsV, diamonds; cpsVI, open circles) were grown in medium alone or increasing concentrations of zinc chloride (125 μM, 250 μM, 500 μM, 750 μM, 1000 μM, 2500 μM, 5000 μM). Bacterial growth was measured at 24 h post-inoculation as an optical density at 600 nm absorbance (OD_600_). At 125, 250, 500, 750, 1000 and 2500 μM concentrations of zinc, cpsIII strains of GBS (inverted triangles) exhibited less susceptibility to zinc intoxication than other capsular serotypes, as determined by one-way ANOVA with Tukey’s post hoc multiple correction (*P < 0.05, **P < 0.01, ***P < 0.001, ****P < 0.0001, *n* = 3 biological replicates)

Capsular serotypes have been implicated as an important virulence factor that aids in evasion of the innate immune response [21]. Additionally, capsular serotype III strains are associated with higher rates of invasive neonatal disease [22] and account for the majority of late-onset meningitis cases in neonates [23]. Because invasive strains and isolates from EOD were less susceptible to zinc toxicity than rectovaginal colonizing strains, we hypothesized that capsular serotype variation could contribute to alterations in susceptibility to zinc toxicity. To test this, we stratified strains based on capsular serotype and analyzed growth in cultures exposed to increasing concentration of zinc (Fig. 5). At concentrations as low as 125 μM of zinc, capsular serotype III (cpsIII) strains emerged as having enhanced growth compared to the cpsIb and cpsII strains. Notably, the cpsIII strains also exhibited enhanced growth at 250, 500, 750, 1000, and 2500 μM zinc compared to the cpsIb strains (*P* < 0.05, one-way ANOVA). At 2500 μM zinc, cpsIII strains exhibited the highest mean growth (mean OD_600_ = 0.267), followed by strains with cpsIa (mean OD_600_ = 0.246), cpsV (mean OD_600_ = 0.200), cpsII (mean OD_600_ = 0.126), and cpsIb (mean OD_600_ = 0.107). The cpsVI strains were most sensitive to zinc toxicity at a concentration of 2500 μM (mean OD_600_ = 0.100) compared to other capsular serotypes. However, at concentrations of 125, 250, 500, 750, and 1000 μM zinc, cpsIb isolates consistently exhibited the lowest growth, or greatest level of inhibition, among all capsular serotypes tested, underscoring their susceptibility to zinc intoxication (*P* < 0.05, one-way ANOVA). At a concentration of 5000 μM zinc, no statistically significant differences were observed between capsular types, a result that is likely due to a threshold effect of all strains experiencing significant growth inhibition.

**Fig. 6:**
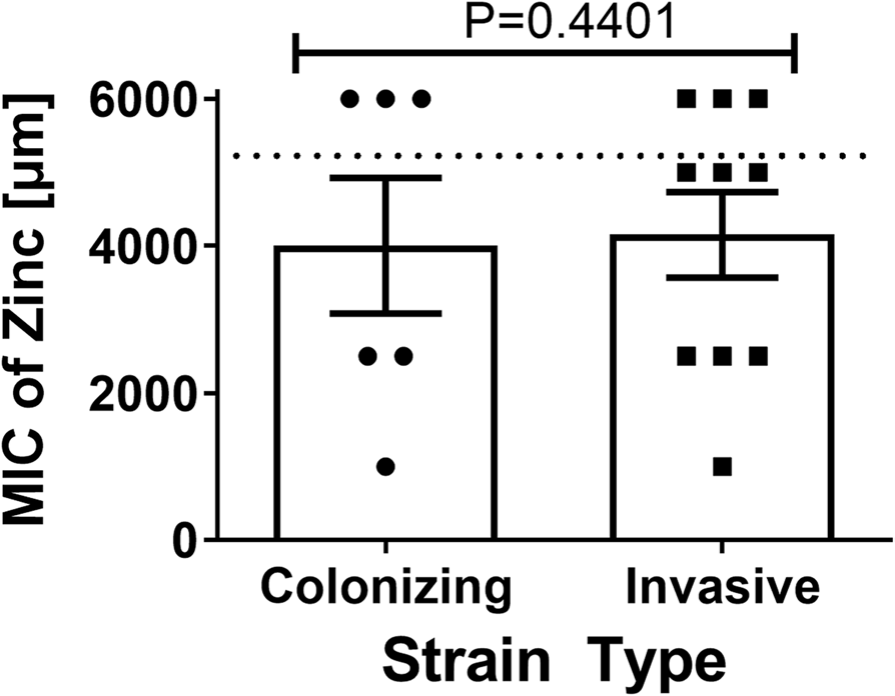
Minimum Inhibitory Concentration (MIC) of zinc chloride required to inhibit GBS growth across colonizing or invasive strains of capsular serotype III (cpsIII) isolates. Analysis of susceptibility to zinc-associated growth inhibition in invasive vs. clinical isolates of Group B *Streptococcus* (GBS). GBS strains isolated from colonized patients, or patients experiencing invasive disease were grown in increasing concentrations of zinc chloride and mean MIC was calculated. Mann-Whitney U test revealed no statistically significant differences between colonizing and invasive strains of the cpsIII cohort (*P* = 0.4401). Dotted line indicates upper limit of detection

Because our results have shown that colonizing strains may be more susceptible to zinc toxicity, and the cpsIII strains exhibited low susceptibility to zinc toxicity, we stratified the cpsIII strains into invasive versus colonizing strains to ascertain if there were any differences in these two cohorts (Fig. 6). Mann-Whitney U analyses revealed no statistically significant difference between invasive and colonizing cpsIII strains (*P* = 0.4401).

### GBS susceptibility to zinc toxicity varies across sequence types

**Fig. 7:**
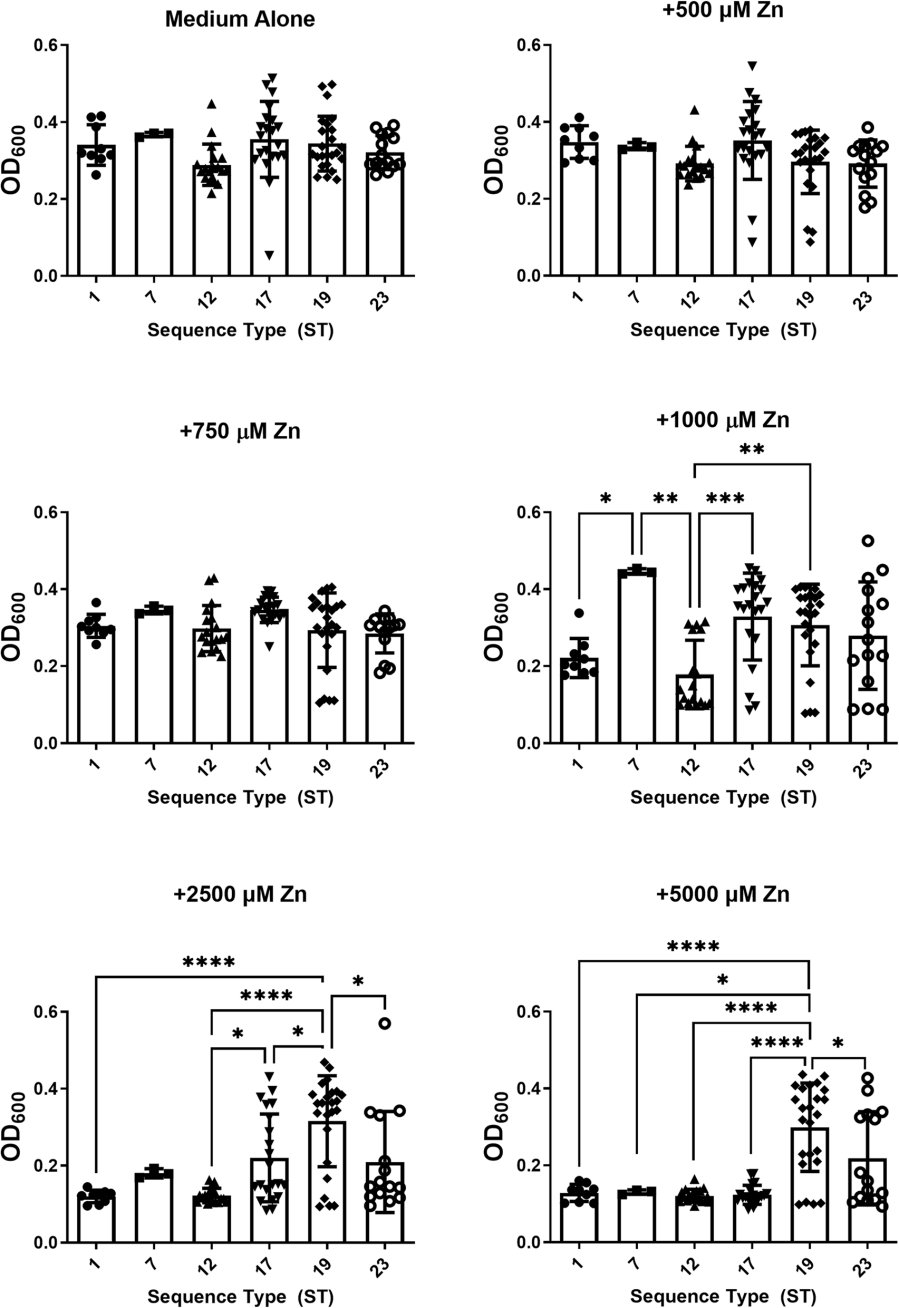
Analysis of susceptibility to growth inhibition by zinc intoxication in diverse sequence types of Group B *Streptococcus* (GBS). GBS strains of varying sequence types (ST-1, black circles; ST-7, squares; ST-12, triangles; ST-17, inverted triangles; ST-19, diamonds; ST-23, open circles) were grown in medium alone or increasing concentrations of zinc chloride (500 μM, 750 μM, 1000 μM, 2500 μM, 5000 μM). Bacterial growth was measured at 24 h post-inoculation as an optical density at 600 nm absorbance (OD_600_). In medium alone and at 500 and 750 μM concentrations of zinc, no differences in susceptibility were observed. However, at 1000, 2500, and 5000 μM zinc concentrations, ST-1 and ST-7 strains exhibited the highest susceptibility to zinc intoxication than other sequence types. At 2500 μM zinc concentration, ST-17 and ST-19 emerged as the least susceptible strain types. At 5000 μM zinc concentration, ST-19 remained the least susceptible strain type. Statistical significance was determined by one-way ANOVA with Tukey’s post hoc multiple correction (*P < 0.05, **P < 0.01, ***P < 0.001, ****P < 0.0001, *n* = 3 biological replicates)

Because different GBS sequence types (STs) are associated with maternal colonization and neonatal disease [24], it is possible that strains of different STs have variable mechanisms to facilitate metal homeostasis. To test this hypothesis, we stratified GBS strains by ST and analyzed the growth under varying concentrations of zinc (Fig. 7). At 2500 μM zinc, ST-17, ST-19, and ST-23 remained the least susceptible to zinc intoxication compared to ST-1, ST-7, ST-12 (*P* < 0.05, one-way ANOVA). At 5000 μM zinc, ST-19 remained the least susceptible to zinc intoxication compared to all other strains tested (*P* < 0.05, one-way ANOVA). Interestingly, the ST-19 isolates that exhibited the highest resistance to zinc intoxication were all classified as cpsIII strains.

## Discussion

The battle for essential nutrient metals between the vertebrate host and invading pathogen has been closely linked to virulence [25]. Starvation of transition metals is detrimental to bacteria, however, high concentrations of metals like zinc also have antimicrobial effects [10, 12]. Zinc plays an important antimicrobial role in innate immune defense against several pathogens, including a variety of *Streptococcus* spp. [23]. Bacteria that have the capability to survive and replicate inside the phagosome of macrophages employ and regulate metal transport machinery to maintain metal homeostasis. For example, the notorious intracellular pathogen, *Mycobacterium tuberculosis*, has been shown to upregulate P-type ATPases which act in heavy metal efflux to counteract the toxic, high extracellular zinc levels of the phagosome [26]. In fact, metal intoxication survival is a critical virulence trait in *S. pneumoniae* and *S. pyogenes* [27–30] as well as GBS [14]. In this study, we sought to determine the differences in susceptibility or resistance to zinc toxicity in 30 strains of GBS, spanning diverse capsular serotypes, STs, isolation source, and disease manifestation.

Our work demonstrates that GBS colonizing and invasive strain types differ in susceptibility to zinc intoxication. Specifically, invasive strains exhibit diminished susceptibility to zinc toxicity compared to colonizing strains, indicating invasive strains may have acquired adaptations to survive metal intoxication strategies imposed within the vertebrate host during invasive infections. GBS strains from varying isolation sources also exhibit varying susceptibility to zinc intoxication. In particular, strains isolated from early onset disease manifestations have significantly enhanced tolerance of zinc intoxication compared to rectovaginal colonizing isolates. Again, similar to the results observed with colonizing versus invasive isolates, this could reflect strain-specific adaptation to the ecological niche of the host. The human vaginal environment is rich in S100A-family proteins, especially during infectious processes, and these proteins bind and sequester nutrient metals, such as zinc [31–34]. It is possible that the GBS-colonized vaginal mucosa represents an environment with low zinc availability, thus there is no selective pressure for colonizing strains to develop strategies to circumnavigate high zinc concentrations. Conversely, numerous studies have shown that circulating innate immune cells such as macrophages and neutrophils use zinc intoxication as a strategy to inhibit invading microbes [11, 35–37]. It is possible that invasive strains have undergone selection for survival in high zinc environments, yielding strains with greater resistance to the toxicity of this transition metal.

Our results indicate that GBS capsular serotype also confers varying susceptibility to zinc intoxication. Specifically, cpsIII isolates were less susceptible to zinc intoxication, whereas cpsIb isolates were much more susceptible to zinc toxicity. This is interesting because cpsIII is the predominant capsular serotype responsible for invasive neonatal infections. Additionally, there is growing evidence that bacterial exopolysaccharides have strong binding properties for metal sorption [38, 39]. Additionally, production of specific exopolysaccharides can promote cellular survival of metal stress [40–42]. Thus, it remains possible that the variations in capsular polysaccharide production responsible for alterations in capsular serotype of the surveyed GBS strains could contribute to alternate binding of excess metals, thereby altering GBS susceptibility to intoxication with metals, such as zinc.

Our study also revealed that different sequence types of GBS had varying susceptibilities to zinc intoxication. Specifically, ST-1 and ST-12 were highly susceptible to zinc stress, while ST-17, ST-19, and ST-23 were much more resistant to zinc intoxication. A cross-continental study revealed that GBS ST-1 and ST-19 are associated with asymptomatic colonization, while ST-17 is predominantly associated with invasive neonatal disease [24]. ST-23 was associated with both rectovaginal carriage and invasive GBS disease [24]. By contrast, STs 1, 17, 19 and 23, were all found to colonize pregnant women at higher rates in different patient populations [18]. Additionally, ST-17 (specifically capsular serotype III strains in this clade) was linked to EOD and strongly associated with LOD and meningitis [19, 43, 44]. This finding further supports a model in which invasive strains are likely undergoing positive selection for zinc resistance as a critical virulence factor to overcome innate immune defenses which employ zinc intoxication as an antimicrobial strategy [45]. Interestingly, in our study, ST-19 strains (largely colonizing strains) that were most resistant to zinc intoxication were all cpsIII strains, underscoring the relationship between capsular polysaccharide production and zinc resistance in GBS.

### Limitations of the study

There are several limitations of our study including the clinical definitions of “colonizing” versus “invasive” strains which can be imperfect. Isolating strains from invasive neonatal infections proves that such strains are capable of causing perinatal infection. However, simply because a “colonizing” strain was isolated from a rectal or vaginal swab does not mean it would be incapable of causing invasive disease under different circumstances. Thus, some “colonizing” strains might be misclassified because they have unrecognized invasive potential. Such a misclassification could bias towards a null hypothesis (contributing to a type II error). Additional genomics studies are also warranted to identify genetic traits linked to zinc resistance, particularly in the more virulent lineages. An additional limitation includes the medium sample size which should be expanded in future studies to include more representation in other capsular serotypes and sequence types to draw broader conclusions across a larger number of GBS strains.

## Conclusions

In conclusion, we report strain variations within a cohort of GBS strains with respect to susceptibility to zinc intoxication across STs, capsular serotypes, isolation source, and invasive versus colonizing strains. Invasive isolates demonstrated greater resistance to zinc toxicity compared to colonizing strains. Additionally, ST-1 and ST-12 were highly susceptible to zinc stress, while ST-17, ST-19, and ST-23 were much more resistant to zinc intoxication. cpsIII isolates were less susceptible to zinc intoxication, whereas cpsIb isolates were much more susceptible to zinc toxicity. Our study is a pilot study that is hamstrung by the relatively small number of strains. Future studies will require an expansion to include genetic studies and a larger number of strains and diverse capsular and sequence types, as well as GBS strains from non-perinatal sources and distinct geographic locations.

## Data Availability

The datasets used and/or analyzed during the current study available from the corresponding authors upon reasonable request.

## Abbreviations

GBS: Group B *Streptococcus*
PPROM: preterm prelabor rupture of the gestational membranes
EOD: Early onset disease
LOD: Late onset disease
IAP: Intrapartum antibiotic prophylaxis
Zn^2+^: Zinc
ZnCl_2_: Zinc chloride
ST: Sequence type
TSA: Tryptic soy agar
OD_600_: Optical density at 600 nm
MIC: Minimum inhibitory concentration
cps: Capsular polysaccharide
rpm: Rotations per minute
nm: Nanometer
μM: Micromolar

## References

[1] Patras KA, Nizet V (2018). Group B streptococcal maternal colonization and neonatal disease: molecular mechanisms and preventative approaches. Front Pediatr.

[2] Raabe VN, Shane AL. Group B *Streptococcus* (*Streptococcus agalactiae*). Microbiol Spectr. 2019;7. 10.1128/microbiolspec GPP3-0007-2018. 10.1128/microbiolspec.gpp3-0007-2018 PMC6432937 30900541

[3] Campbell JR, Hillier SL, Krohn MA, Ferrieri P, Zaleznik DF, Baker CJ (2000). Group B streptococcal colonization and serotype-specific immunity in pregnant women at delivery. Obstet Gynecol.

[4] Stoll BJ, Schuchat A (1998). Maternal carriage of group B *streptococci* in developing countries. Pediatr Infect Dis J.

[5] Armistead B, Oler E, Adams Waldorf K, Rajagopal L (2019). The double life of group B *Streptococcus*: asymptomatic colonizer and potent pathogen. J Mol Biol.

[6] Schrag SJ, Verani JR (2013). Intrapartum antibiotic prophylaxis for the prevention of perinatal group B streptococcal disease: experience in the United States and implications for a potential group B streptococcal vaccine. Vaccine..

[7] Nanduri SA, Petit S, Smelser C, Apostol M, Alden NB, Harrison LH, Lynfield R, Vagnone PS, Burszlaff K, Spina NL, Dufort EM, Schaffner W, Thomas AR, Farley MM, Jain JH, Pondo T, McGee L, Beall BW, Schrag SJ (2019). Epidemiology of invasive early-onset and late-onset group B streptococcal disease in the United States, 2006 to 2015: multistate laboratory and population-based surveillance. JAMA Pediatr.

[8] Shipitsyna E, Shalepo K, Zatsiorskaya S, Krysanova A, Razinkova M, Grigoriev A, Savicheva A (2020). Significant shifts in the distribution of vaccine capsular polysaccharide types and rates of antimicrobial resistance of perinatal group B *streptococci* within the last decade in St. Petersburg, Russia. Eur J Clin Microbiol Infect Dis.

[9] Andreini C, Bertini I, Cavallaro G, Holliday GL, Thornton JM (2008). Metal ions in biological catalysis: from enzyme databases to general principles. J Biol Inorg Chem.

[10] Djoko KY, Ong CY, Walker MJ, McEwan AG (2015). The role of copper and zinc toxicity in innate immune defense against bacterial pathogens. J Biol Chem.

[11] Wagner D, Maser J, Lai B, Cai Z, Barry CE, Bentrup HZ, K, Russell DG, Bermudez LE. (2005). Elemental analysis of *Mycobacterium avium*-, *mycobacterium tuberculosis*-, and *mycobacterium smegmatis*-containing phagosomes indicates pathogen-induced microenvironments within the host cell’s endosomal system. J Immunol.

[12] Stafford SL, Bokil NJ, Achard MES, Kapetanovic R, Schembri MA, McEwan AGS, et al. Metal ions in macrophage antimicrobial pathways: emerging roles for zinc and copper. Biosci Rep. 2013;33. 10.1042/BSR20130014. 10.1042/BSR20130014 PMC3712485 23738776

[13] Hood MI, Skaar EP (2012). Nutritional immunity: transition metals at the pathogen–host interface. Nat Rev Microbiol.

[14] Sullivan MJ, Goh KGK, Ulett GC. Cellular Management of Zinc in Group B *Streptococcus* Supports Bacterial Resistance against Metal Intoxication and Promotes Disseminated Infection. mSphere. 2021;6. 10.1128/mSphere.00105-21. 10.1128/mSphere.00105-21 PMC8265624 34011683

[15] Bonaventura P, Benedetti G, Albarède F, Miossec P (2015). Zinc and its role in immunity and inflammation. Autoimmun Rev.

[16] Davies HD, Adair C, McGreer A, Ma D, Robertson S, Mucenski M, Kowalsky L, Tyrell G, Baker CJ (2001). Antibodies to capsular polysaccharides of group B *Streptococcus* in pregnant Canadian women: relationship to colonization status and infection in the neonate. J Infect Dis.

[17] Spaetgens R, DeBella K, Ma D, Robertson S, Mucenski M, Davies HD. Perinatal antibiotic usage and changes in colonization and resistance rates of group B streptococcus and other pathogens. 2002;100:525–33. 10.1016/s0029-7844(02)02068-9. 10.1016/s0029-7844(02)02068-9 12220773

[18] Manning SD, Springman AC, Lehotzky E, Lewis MA, Whittam TS, Davies HD (2009). Multilocus sequence types associated with neonatal group B streptococcal sepsis and meningitis in Canada. J Clin Microbiol.

[19] Manning SD, Lewis MA, Springman AC, Lehotzky E, Whittam TS, Davies HD (2008). Genotypic diversity and serotype distribution of group B *streptococcus* isolated from women before and after delivery. Clin Infect Dis.

[20] Lu J, Francis JD, Guevara MA, Moore RE, Chambers SA, Doster RS, Eastman AJ, Rogers LM, Noble KN, Manning SD, Damo SM, Aronoff DM, Townsend SD, Gaddy JA (2021). Antibacterial and anti-biofilm activity of the human breast Milk glycoprotein Lactoferrin against group B *Streptococcus*. Chembiochem..

[21] Rajagopal L (2009). Understanding the regulation of group B streptococcal virulence factors. Future Microbiol.

[22] Alhhazmi A, Pandey A, Tyrrell GJ (2017). Identification of group B *Streptococcus* capsule type by use of a dual phenotypic/genotypic assay. J Clin Microbiol.

[23] Bellais S, Six A, Fouet A, Longo M, Dmytruk N, Glaser P, Trieu-Cuot PC (2012). Capsular switching in group B *Streptococcus* CC17 hypervirulent clone: a future challenge for polysaccharide vaccine development. J Infect Dis.

[24] Jones N, Bohnsack JF, Takahashi S, Oliver KA, Chan M-S, Kunst F, Glaser P, Rusniok C, Crook DWM, Harding RM, Bisharat N, Spratt BG (2003). Multilocus sequence typing system for group B *streptococcus*. J Clin Microbiol.

[25] Palmer LD, Skaar EP (2016). Transition metals and virulence in Bacteria. Annu Rev Genet.

[26] Botella H, Peyron P, Levillain F, Poincloux R, Poquet Y, Brandli I, Wang C, Tailleux L, Tilleul S, Charriere GM, Waddell SJ, Foti M, Lugo-Villarino G, Gao Q, Maridonneau-Parini I, Butcher PD, Castagnoli PR, Gicquel B, de Chastellier C, Neyrolles O (2011). Mycobacterial p(1)-type ATPases mediate resistance to zinc poisoning in human macrophages. Cell Host Microbe.

[27] Shafeeq S, Kuipers OP, Kloosterman TG (2013). The role of zinc in the interplay between pathogenic *streptococci* and their hosts. Mol Microbiol.

[28] Turner AG, Ong C-LY, Walker MJ, Djoko KY, McEwan AG (2017). Transition metal homeostasis in *Streptococcus pyogenes* and *Streptococcus pneumoniae*. Adv Microb Physiol.

[29] Danilova TA, Danilina GA, Adzhieva AA, Vostrova EI, Zhukhovitskii VG, Cheknev SB (2020). Inhibitory effect of copper and zinc ions on the growth of *Streptococcus pyogenes* and *Escherichia coli* biofilms. Bull Exp Biol Med.

[30] Makthal N, Kumaraswami M (2017). Zinc’ing it out: zinc homeostasis mechanisms and their impact on the pathogenesis of human pathogen group a *Streptococcus*. Metallomics..

[31] Leizer J, Nasioudis D, Forney LJ, Schneider GM, Gliniewicz K, Boester A, Witkin SS (2018). Properties of epithelial cells and vaginal secretions in pregnant women when *lactobacillus crispatus* or *lactobacillus iners* dominate the vaginal microbiome. Reprod Sci.

[32] Yano J, Lilly E, Barousse M, Fidel PL (2010). Epithelial cell-derived S100 calcium-binding proteins as key mediators in the hallmark acute neutrophil response during Candida vaginitis. Infect Immun.

[33] Haley KP, Delgado AG, Piazuelo MB, Mortensen BL, Correa P, Damo SM, Chazin WJ, Skaar EP, Gaddy JA (2015). The human antimicrobial protein calgranulin C participates in control of *helicobacter pylori* growth and regulation of virulence. Infect Immun.

[34] Damo SM, Kehl-Fie TE, Sugitani N, Holt ME, Rathi S, Murphy WJ, Zhang Y, Betz C, Hench L, Fritz G, Skaar EP, Chazin WJ (2013). Molecular basis for manganese sequestration by calprotectin and roles in the innate immune response to invading bacterial pathogens. Proc Natl Acad Sci U S A.

[35] Neyrolles O, Wolschendorf F, Mitra A, Niederweis M (2015). Mycobacteria, metals, and the macrophage. Immunol Rev.

[36] Neyrolles O, Mintz E, Catty P (2013). Zinc and copper toxicity in host defense against pathogens: *mycobacterium tuberculosis* as a model example of an emerging paradigm. Front Cell Infect Microbiol.

[37] von Pein JB, Stocks CJ, Schembri MA, Kapetanovic R, Sweet MJ (2021). An alloy of zinc and innate immunity: Galvanising host defence against infection. Cell Microbiol.

[38] Maalej H, Hmidet N, Boisset C, Buon L, Heyraud A, Nasri M (2015). Optimization of exopolysaccharide production from *pseudomonas stutzeri* AS22 and examination of its metal-binding abilities. J Appl Microbiol.

[39] De Philippis R, Colica G, Micheletti E (2011). Exopolysaccharide-producing cyanobacteria in heavy metal removal from water: molecular basis and practical applicability of the biosorption process. Appl Microbiol Biotechnol.

[40] Mukherjee P, Mitra A, Roy M (2019). *Halomonas* Rhizobacteria of *Avicennia marina* of Indian Sundarbans promote Rice growth under saline and heavy metal stresses through exopolysaccharide production. Front Microbiol.

[41] Polak-Berecka M, Szwajgier D, Waśko A (2014). Biosorption of Al(+3) and cd(+2) by an exopolysaccharide from *lactobacillus rhamnosus*. J Food Sci.

[42] Mitra A, Chatterjee S, Kataki S, Rastogi RP, Gupta DK. Bacterial tolerance strategies against lead toxicity and their relevance in bioremediation application. Environ Sci Pollut Res Int 2021;28:14271–14284. oi: 10.1007/s11356-021-12583-9. 10.1007/s11356-021-12583-9 33528774

[43] Musser JM, Mattingly SJ, Quentin R, Goudeau A, Selander RK (1989). Identification of a high-virulence clone of type III *Streptococcus agalactiae* (group B *Streptococcus*) causing invasive neonatal disease. Proc Natl Acad Sci U S A.

[44] Lin F-YC, Whiting A, Adderson E, Takahashi S, Dunn DM, Weiss R, Azimi PH, Phillips JB, Weisman LE, Regan J, Clark P, Rhoads GG, Frasch CE, Troendle J, Moyer P, Bohnsack JF (2006). Phylogenetic lineages of invasive and colonizing strains of serotype III group B *streptococci* from neonates: a multicenter prospective study. J Clin Microbiol.

[45] Ong CY, Gillen CM, Barnett TC, Walker MJ, McEwan AG (2014). An antimicrobial role for zinc in innate immune defense against group a *streptococcus*. J Infect Dis.

